# Isolation and characterization of *Yersinia* phage fMtkYen3-01

**DOI:** 10.1007/s00705-024-06149-6

**Published:** 2024-10-19

**Authors:** Sophia Goladze, Sheetal Patpatia, Henni Tuomala, Matti Ylänne, Nino Gachechiladze, Daniel de Oliveira Patricio, Mikael Skurnik, Lotta-Riina Sundberg

**Affiliations:** 1 Department of Biological and Environmental Science and Nanoscience Center, University of Jyväskylä, Jyväskylä, Finland; 2 Human Microbiome Research Program, Department of Bacteriology and Immunology, Faculty of Medicine, University of Helsinki, Helsinki, Finland; 3 Faculty of Exact and Natural Sciences, Ivane Javakhishvili Tbilisi State University, Tbilisi, Georgia

## Abstract

*Yersinia enterocolitica* causes yersiniosis, the third most common gastrointestinal infection in humans throughout Europe. The emergence of multidrug resistance and the lack of effective new antibiotics have drawn attention to phage therapy as a treatment option. Here, we report the complete genome sequence of phage fMtkYen3-01, which infects *Y. enterocolitica* serotype O:3 strains. This phage has a genome 40,415 bp in length with 45.1% GC content and 49 predicted genes. fMtkYen3-01 infected 9.5% of the 42 *Y. enterocolitica* strains tested and showed stability at 25–40 °C, as well as pH 5.0–10.0. These results suggest the therapeutic potential of this phage.

**Supplementary Information:**

The online version contains supplementary material available at 10.1007/s00705-024-06149-6.

*Yersinia enterocolitica* is a highly heterogeneous species of Gram-negative enteric bacteria. Some sero/biotypes are pathogenic and are the third most common etiologic agents of foodborne zoonoses in the EU/EEA [1, 2]. In 2020, the prevalence of yersiniosis was 1.8 cases per 100,000 population, with the highest rates in children under 4 years [1, 3]. *Y. enterocolitica* strains are classified into six biotypes: avirulent (biotype 1A), highly pathogenic (biotype 1B), and weakly pathogenic (biotypes 2–5), with biotype 4/serotype O:3 strains being the most widespread in Europe [4]. Clinical manifestation of yersiniosis may vary from self-limiting mild diarrhea to severe gastroenteritis, sometimes followed by reactive arthritis (ReA) or erythema nodosum, depending on age, comorbidities, and the immune status of the patient [5, 6]. Considering the substantial impact of *Y. enterocolitica* infection on public health and the increasing spread of multidrug-resistant bacterial pathogens, alternative treatment strategies are urgently needed [7].

Bacteriophages (phages) are bacterial viruses that have been recognized as a promising tool for biotherapeutic and biocontrol applications for more than a century [8–10]. If new phage isolates are properly screened for lytic efficiency and genomic safety, they can be a potent alternative to antibiotics and synthetic antiseptic agents [11].

Here, we report the characterization of a new bacteriophage, fMtkYen3-01, isolated from the Mtkvari River in Georgia (41.68999, 44.81029) using *Y. enterocolitica* serotype O:3 strain 6471/76-c (YeO3-c) as an enrichment host. The *Yersinia* host was cultured in lysogeny broth (LB) (10 g of tryptone, 5 g of yeast extract, and 10 g of NaCl per liter), supplemented with 0.4% or 1.5% agar, as needed. All incubations were done at 25°C. Plaques were purified three times, followed by counting and propagation using a double-layer agar assay [12].

The *in vitro* infectivity of fMtkYen3-01 was determined using a time-kill assay as described previously [13–15], using a Bioscreen C plate reader (Growth Curves AB Ltd, Helsinki, Finland). In order to assess their antibacterial efficiency across a broad spectrum of clinically relevant *Yersinia* strains, 42 human isolates were selected, representing four *Yersinia* species and 18 serotypes (Supplementary Table S1). Ten µL of phage lysate containing 10^8^ plaque-forming units (PFU) /mL was added to the wells of a honeycomb plate containing 200 µL of 1:20-diluted overnight host culture, resulting to an approximate multiplicity of infection (MOI) of 0.1. Wells with bacteria only were used as growth controls, and wells with sterile LB served as negative controls. The experiment was performed in triplicate. Plates were incubated at 25°C with continuous shaking for 10 h, and the OD_600_ was measured every 30 min. The mean optical density and standard deviation (SD) were calculated for each sample at each time point. Phage effectiveness was evaluated by calculating the percent reduction in the OD_600_ of the bacterial culture after phage infection, relative to the positive control (Supplementary Table S2). The phage was considered "efficient" if the OD_600_ value of the phage-treated culture was less than 50% of the control, "partially infectious" if the difference between the absorbance values fell between 50% and 70%, and "noninfectious" if it exceeded 70% [13]. Data analysis was done in GraphPad Prism 10.1.0 (GraphPad Software Inc.; San Diego, CA, USA). Phage fMtkYen3-01 demonstrated *in vitro* lytic efficiency against four (9.5%) of the 42 strains tested, showing the highest activity against serotypes O:3 and O:6 (Fig. 1).

**Fig. 1:**
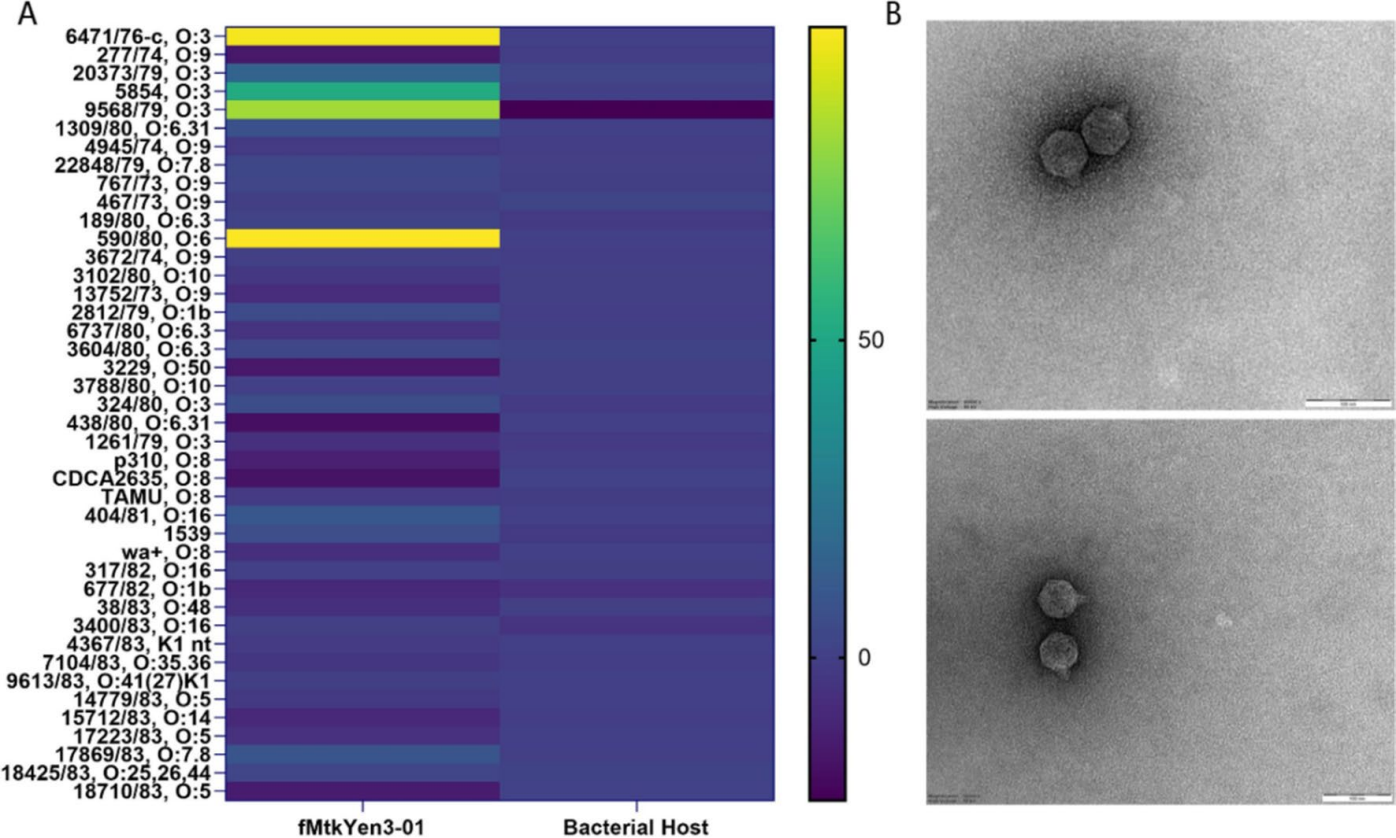
Host range analysis using 42 *Yersinia* strains, representing four species and 18 serotypes. (A) Heat map showing phage lytic activity in liquid cultures, tested by continuous OD_600_ measurement for 10 h. The color code indicates the percent reduction in the OD_600_ value of the phage-infected bacterial host, compared to the uninfected control. (B) Transmission electron micrographs of phage fMtkYen3-01 (x50,000; bar, 100 nm)

The efficiency of plating (EOP) was determined for the four *Yersinia* strains susceptible to fMtkYen3-01. For this purpose, 200 µL of each overnight grown bacterial culture was mixed with 3 mL of 0.4% LB agar and poured onto a 1.5% LB agar plate. After the top agar solidified, 10-µL drops of 10^0^–10^6^ PFU/mL phage lysates were spotted onto the bacterial lawn. The plate was incubated at 25 °C for 24 h, and the EOP was calculated as the ratio of the PFU on the target bacterium to the PFU on the primary host strain. The results showed that phage fMtkYen3-01 had an efficiency of plating within the range of “high production” (EOP ≥ 0.5) for three of the strains tested (Supplementary Fig. S1).

Since lipopolysaccharide (LPS) is a typical virulence factor and a receptor for phage adsorption in Gram-negative bacteria [16–18], we hypothesized that mutations in LPS could influence phage sensitivity. In wild-type serotype O:3 and O:9 bacteria, the LPS is composed of lipid A (LA) substituted with a heptose-rich inner core (IC), half of which is ligated to the outer core hexasaccharide (OC) and the other half to the O-polysaccharide (Oag) [17]. We screened a set of 27 engineered O:3 and O:9 strains with different LPS phenotypes (Supplementary Table S1 and Supplementary Fig. S2). While the phage does not absolutely need Oag or OC, as elimination of one or the other decreases the EOP to 0.22 (for YeO3-R1) or 0.14 (for YeO3-c-OC), elimination of both in YeO3-c-OCR completely knocks out the receptor. The YeO3-R1-M164, -M195, and -M205 mutants, which were resistant to the phage, also lack both Oag and OC, as they carry truncated IC. This indicates that the phage needs an LA-IC substituted with either Oag or OC, and for full infectivity, with both.

The *Y. enterocolitica* O:3 LPS oligosaccharide and polysaccharide ligase mutants showed lowered EOPs. Also in these mutants, elimination of Oag and OC at the same time significantly reduced, but did not completely abolish, recognition by phage fMtkYen3-01 (Supplementary Fig. S2). While wild-type serotype O:9 bacteria were generally not infected by fMtkYen3-01 (Supplementary Table S1), elimination of the O:9 Oag (YeO9-OCR) rendered these bacteria fully sensitive to the phage (Supplementary Fig. S2).

As the first sugar residue in both Oag and OC is *N-*acetylhexosamine [17], it is possible that the *N*-acetyl residue of the first sugar together with IC heptose(s) could be part of the receptor. In LPS biosynthesis, both Oag and OC need a ligase to generate the glycoside bond between the *N*-acetyl sugar and the IC heptose [17]. In mutants lacking Oag or OC, the ligases add only the *N-*acetylhexosamine residue, resulting in a single sugar residue in all of the mutants except the triple ligase mutant YeO3-c-Lps-Los-Lxs, which had the lowest EOP (Supplementary Fig. S2).

Phage stability under different environmental conditions was tested under various pH and temperature conditions. Briefly, 100 µL of phage (1 × 10^6^ PFU/mL) was mixed with 900 µL of LB that had been adjusted to pH 2.0–12.0 using 1 M NaOH or 1 M HCl and incubated at 25 °C for 3 h. Similarly, survival of virions under different temperature conditions was evaluated by inoculating 100 µL of the phage sample (1 × 10^6^ PFU/mL) into 900 µL of LB and incubating at 25, 37, 40, or 65 °C for 3 h, followed by titration. All assays were performed in triplicate. Phage titers were determined using a drop test. PFU counts obtained at pH 7.0 and 25 °C were used as reference values. The assay showed that the phage maintained stability within a pH range of 5.0 to 11.0 and at temperatures between 25°C and 40°C for 3 h without substantial loss of viability (Supplementary Fig. S3).

Phage purification was performed using PEG precipitation, followed by glycerol gradient ultracentrifugation as described previously [19]. Purified phage particles were sedimented onto carbon-coated copper grids and negatively stained with 2% uranyl acetate. fMtkYen3-01 morphology was studied using a JEOL JEM-1400 transmission electron microscope (JEOL Ltd., Tokyo, Japan). Morphological analysis showed that phage fMtkYen3-01 has a 54 ± 0.2 nm (n = 10) capsid and a 14 ± 0.3 nm (n = 10) tail. These characteristics indicate that this phage is a member of the class *Caudoviricetes* with podovirus morphology.

Phage DNA was extracted from a 4 × 10^9^ PFU/mL lysate, using a Maxwell RSC Viral TNA Purification Kit (Promega Corporation, Madison, WI, USA) according to the manufacturer’s instructions. DNA samples were quantified using an Invitrogen Qubit dsDNA BR Assay Kit (Thermo Fisher Scientific, USA), and their integrity and purity were examined by electrophoresis in a 1% agarose gel. Phage DNA was sequenced on an Illumina HiSeq platform at Novogene (Cambridge, UK), using a 150-bp paired-end protocol. The phage genome sequence was assembled *de novo* using the A5 pipeline (Version A5-miseq 20160825), with 250,000 raw reads, resulting in 35x sequence coverage. DNA termini and phage packaging mechanisms were evaluated using PhageTerm [20]. Initial annotation of the genome was done using the Rapid Annotation Server using Subsystem Technology (RAST) [21]. Sequence similarity searches and predictions of protein functions were performed using BLASTp [22], HHpred [23], and HMMER [24]. PHASTER (Phage Search Tool Enhanced Release) was used as an additional tool for phage sequence annotation [25]. tRNA genes were identified using tRNAscan-SE version 2.0 [26]. A phylogenetic tree was generated using Geneious, version 2023.0.4, and a viral proteomic tree was constructed using ViPTree server, version 3.6 [27]. PhageClouds was used to visualize intergenomic relationships between fMtkYen3-01 and closely related phages from the NCBI-GenBank database (threshold of 0.2) [28]. Intergenomic comparison at the nucleotide level was performed using Virus Intergenomic Distance Calculator (VIRIDIC) [29]. Comprehensive Antibiotic Resistance Database (CARD) 3.2.8 and Resistance Gene Identifier (RGI) 6.0.3 were used to detect antibiotic resistance genes.

Phage fMtkYen3-01 has a dsDNA genome of 40,415 bp and 45.1% GC content. PhageTerm analysis revealed 198-bp direct terminal repeats (DTRs) with redundant ends. Annotation yielded 49 protein-coding genes (Supplementary Table S3), with functional assignments for 32 of them. These 32 annotated genes encode structural, DNA replication and packaging, and host-cell-lysis-associated proteins (Supplementary Fig. S4). No tRNA-encoding genes were identified. Annotation did not reveal antibiotic resistance genes, repressors, integrases, or any other genes associated with a temperate life cycle. Furthermore, PhageAI analysis classified fMtkYen3-01 as a virulent phage with prediction accuracy of 100%, highlighting its therapeutic potential.

VIRIDIC showed that the sequence identity of fMtkYen3-01 to related *Yersinia* phages (Supplementary Table S4) belonging to the genus *Helsettvirus*, family *Autographiviridae*, was within the range of 74.3–75.0% (Fig. 2), suggesting that fMtkYen3-01 represents a new species in the same genus. ViPtree analysis based on phage proteomes also provided supporting evidence that fMtkYen3-01 belongs to the family *Autographiviridae*, clustering with the *Pseudomonadota* host group (Fig. 3). Based on PhageClouds analysis, fMtkYen3-01 was connected to 16 *Yersinia* phages belonging to the family *Autographiviridae* that were isolated from fecal samples collected from Finnish pig farms [30] (Supplementary Fig. S5).

**Fig. 2:**
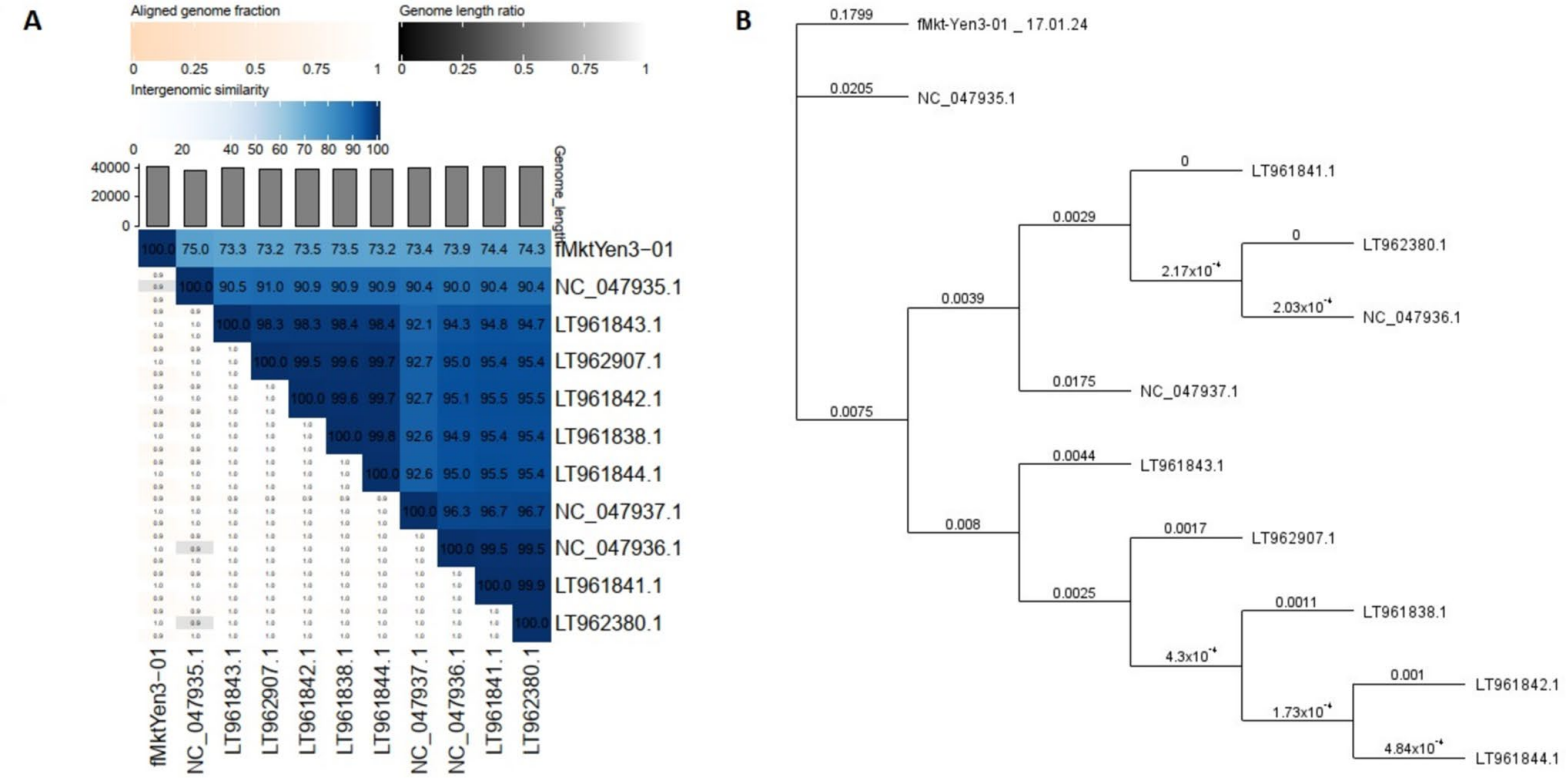
(A) Intergenomic similarity analysis of fMtkYen3-01 and the most closely related phages, calculated using Virus Intergenomic Distance Calculator (VIRIDIC). Horizontal and vertical coordinates correspond to the phage GenBank accession numbers. (B) Phylogenetic neighbor-joining tree based on amino acid sequences of viral proteins of fMtkYen3-01 and 10 closely related phages, obtained from the NCBI database. The tree was generated using Geneious 2023.0.4. LT962907, fPS-10; LT961838, fPS-19; LT961844, fPS-21; LT961843, fPS-50; NC047936, fPS-53; NC047937, fPS-54-ocr; NC047935, fPS-59; LT962380, fPS-85; LT961842, fPS-86; LT961841, fPS-89

**Fig. 3:**
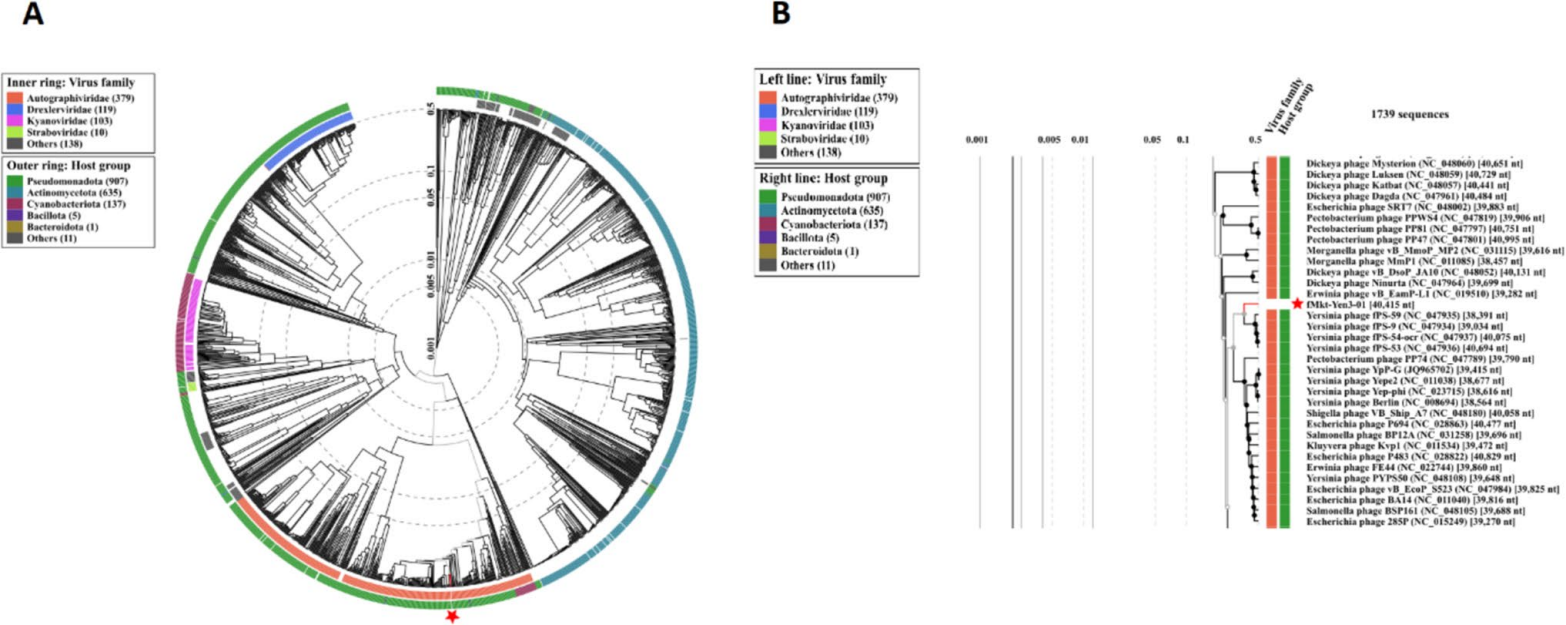
Proteomic tree of phage fMtkYen3-01, generated using ViPTree server version 3.6 based on genome-wide sequence similarities, computed by tBLASTx. (A) ViPtree of fMtkYen3-01 and 1739 other phage genomes. The outer ring indicates the phylum of the bacterial host, while the inner ring indicates phage family classification. (B) Rectangular phylogenetic tree. The inner nodes connect the phages at the subtree level.

Whole-genome-based phylogenetic analysis demonstrated a common evolutionary relationship between fMtkYen3-01 and *Yersinia* fPS phages isolated from Finnish pigs, of which fPS-59 (GenBank accession number NC_047935.1) was the closest one, indicating the wide geographic distribution of phages of the family *Autographiviridae*.

In conclusion, we isolated a virulent phage, fMtkYen3-01, representing a new species within the genus *Helsettvirus,* family *Autographiviridae,* class *Caudoviricetes*. The phage exhibited high *in vitro* efficiency against the isolation host and proved to be stable under various environmental conditions. Based on preliminary experiments, it is likely that the phage uses Oag and OC of LPS as receptors for host recognition and adsorption. Based on the bioinformatic analysis, fMtkYen3-01 was classified as a virulent phage, and no antibiotic resistance, toxic genes, repressors, or integrases were identified in its genome. The results obtained in the study suggest that phage fMtkYen3-01 has therapeutic potential.

## Supplementary Information

Below is the link to the electronic supplementary material.

Supplementary file1 (DOCX 752 KB)

Supplementary file2 (FASTA 40 KB)

## Data Availability

The raw data for phage genome sequencing are available in the SRA database under accession number PRJNA1117053.
