# Supplementary material for: Isolation and characterization of Yersinia phage fMtkYen3-01
Source: Arch Virol. 2024 Oct 19;169(11):226. doi: 10.1007/s00705-024-06149-6 (PMC11490452; doi:10.1007/s00705-024-06149-6)
Supplement: Supplementary file 1 — Supplementary file1 (DOCX 752 KB) [file 705_2024_6149_MOESM1_ESM.docx]

**Supplementary materials**

**Isolation and characterization of *Yersinia* phage fMtkYen3-01**

Sophia Goladze^a,b,c^, Sheetal Patpatia^b^, Henni Tuomala^b^, Matti Ylänne^b^, Nino Gachechiladze^c^, Daniel de Oliveira Patricio^a^, Mikael Skurnik^b^, Lotta-Riina Sundberg^a^

*^a^Department of Biological and Environmental Science and Nanoscience Center, University of Jyväskylä, Jyväskylä, Finland*

*^b^Human Microbiome Research Program, Department of Bacteriology and Immunology, Faculty of Medicine, University of Helsinki, Helsinki, Finland*

*^c^Ivane Javakhishvili Tbilisi State University, Faculty of Exact and Natural Sciences, Tbilisi, Georgia*

*Corresponding Author’s e-mail address:* [*lotta-riina.sundberg@jyu.fi*](mailto:lotta-riina.sundberg@jyu.fi)

**Supplementary Fig. S1** Phage fMtkYen3-01 efficiency of plating (EOP) values on susceptible *Yersinia enterocolitica* strains.


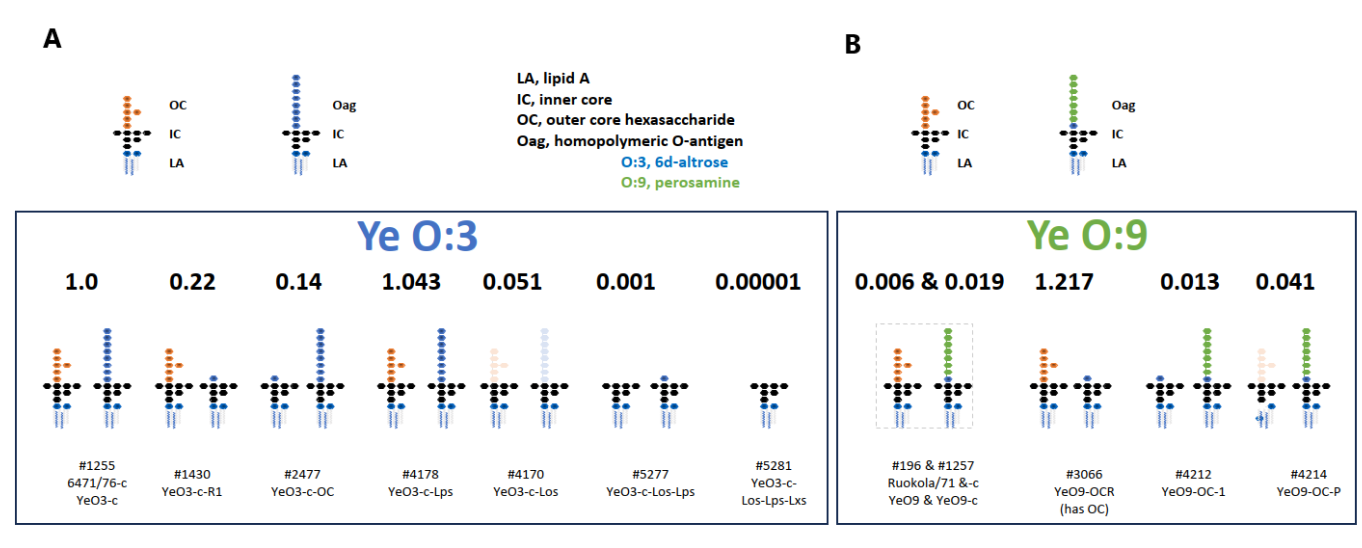


**Supplementary Fig. S2** Phage fMtkYen3-01 EOP-profiles on LPS mutant strains of *Y. enterocolitica* serotypes O:3 and O:9. The schematic LPS phenotypes of the different LPS-mutants of *Y. enterocolitica* serotype O:3 (**A**) and O:9 (**B**) are indicated as well as their respective EOPs.

**
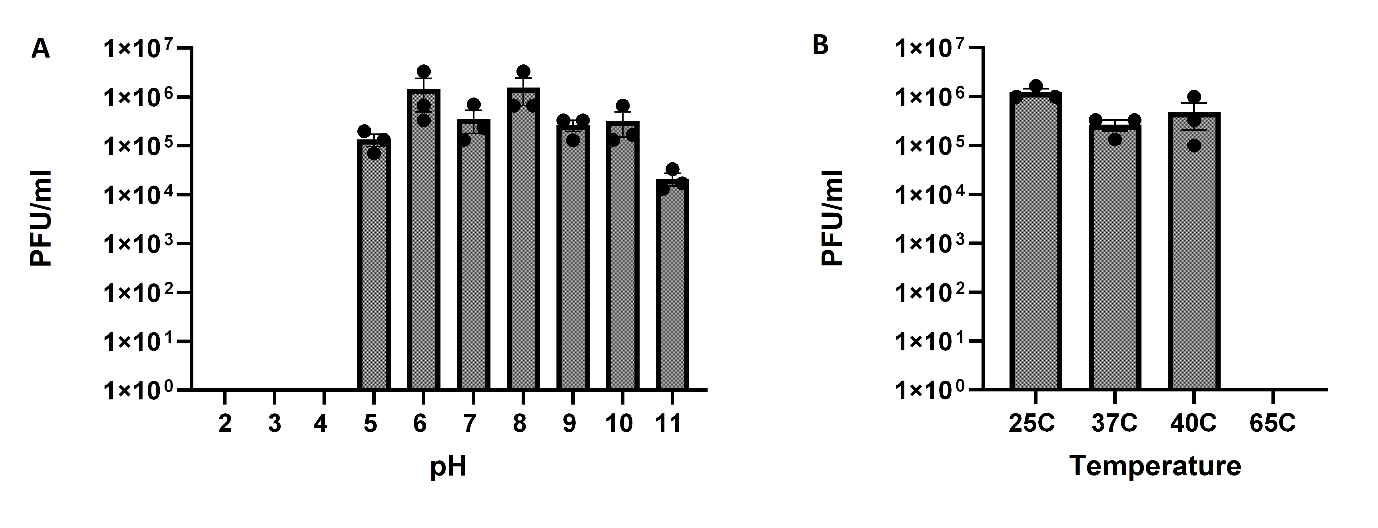
**

**Supplementary Fig. S3** Stability of phage fMtkYen3-01during 3h-incubation under various pH (**A**) and temperature conditions (**B**).


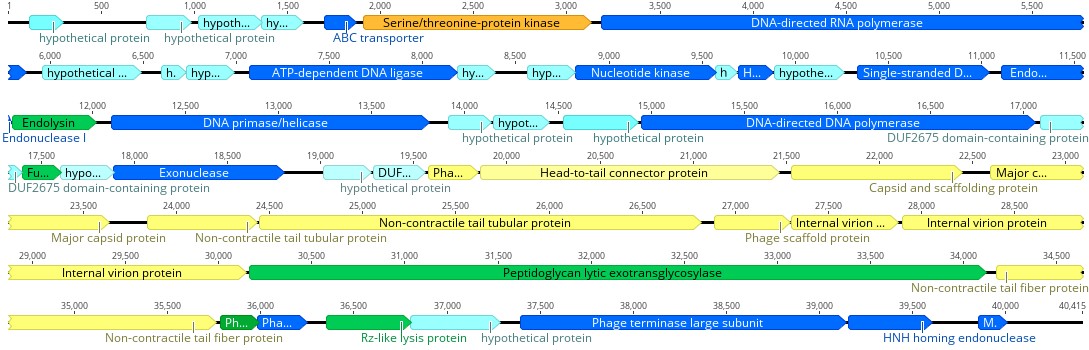


**Supplementary Fig. S4** Genomic organization of phage fMtkYen3-01. Gene orientations are indicated with arrows. Color code is used to mark respective functional predictions.

Blue - DNA metabolism, transcription, translation; green - host cell lysis, viral entry into the host cell; yellow, structural, assembly; orange - protein phosphorylation; turquoise - hypothetical proteins. The graph was generated using Geneious version 2023.0.4.

**
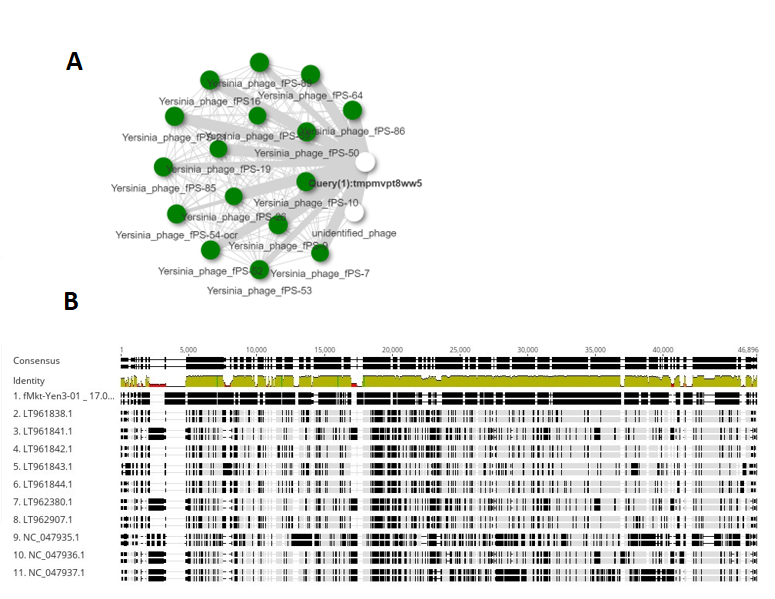
**

**Supplementary Fig. S5** (**A**) Intergenomic relationship between the query phage (fMtkYen3-01) and 16 best reference matches, targeting *Yersinia* spp. The analysis was performed using PhageClouds with intergenomic distance threshold of 0.2. (**B**) Alignment of fMtkYen3-01 with 10 of its closest related phage genomes (Table S4) using Clustal Omega 1.2.2. Green lines indicate 100% sequence identity, brown – 30% to 100%, and red – below 30% identity.

**Supplementary Table S1.** Bacterial strains used in the study and their sensitivity to phage fMtkYen3-01

| **Storage No.** | **Strain Code** | **Serotype** | **Source / Reference** | **References** | **Phage sensitivity (EOP)** |
| --- | --- | --- | --- | --- | --- |
| *Y. aleksiciae* | | | | | |
| 284 | 404/81 | 16 | Human | (1) | - |
| 390 | 317/82 | 16 | Human | Skurnik lab strain collection | - |
| *Y. enterocolitica* | | | | | |
| 286 | 1539 |  | Human | Skurnik lab strain collection | - |
| 533 | 4367/83 | K1 nt | Human | Skurnik lab strain collection | - |
| 2 | 20373/79 | 3 | Human | Skurnik lab strain collection | + (0.3) |
| 3 | 5854 | 3 | Human | Skurnik lab strain collection | + (0.6) |
| 4 | 9568/79 | 3 | Human | Skurnik lab strain collection | + (0.5) |
| 1255 | 6471/76-c (YeO3-c) | 3 | pYV- | (2) | + (1.0) |
| 1430 | YeO3-c-R1 | 3 | Spontaneous rough derivative of 6471/76-c | (3) | + (0.22) |
| 2477 | YeO3-c-OC | 3 | Δ(*wzx-wbcQ*) derivative of YeO3-c | (4) | + (0.14) |
| 2684 | YeO3-c-OCR | 3 | Spontaneous rough derivative of YeO3-c-OC | (4) | - |
| 3118 | YeO3-c-R1-M205 | 3 | *hldE*::Cat-Mu derivative of YeO3-c-R1 | (5) | - |
| 3201 | YeO3-c-R1-M195 | 3 | *gaIU*::Cat-Mu derivative of YeO3-c-R1 | (5) | - |
| 3238 | YeO3-c-R1-M164 | 3 | *waaF*::Cat-Mu derivative of YeO3-c-R1 | (5) | - |
| 7506 | YeO3-R1-15R | 3 | *btuB*::Cat-Mu derivative of YeO3-c-R1 | (6) | + (0.16) |
| 4170 | YeO3-c-Los | 3 | *ΔwaaL*_os_ | (7) | + (0.051) |
| 4178 | YeO3-c-Lps | 3 | *ΔwaaL*_ps_ | (7) | + (1.043) |
| 5277 | YeO3-c-Los-Lps | 3 | *ΔwaaL*_os_ *ΔwaaL*_ps_ | (7) | + (0.001) |
| 5281 | YeO3-c- Los-Lps-Lxs | 3 | *ΔwaaL*_os_ *ΔwaaL*_ps_ *ΔwaaL*_xs_ | (7) | + (0.00001) |
| 6149 | YeO3-R1-Cat17 | 3 | *ompF*::Cat-Mu derivative of YeO3-R1 | (8) | + (0.9) |
| 664 | 14779/83 | 5 | Human | (9) | - |
| 1834 | 14779/83-φR1-37-R | 5 | Spontaneous φR1-37 resistant derivative of 14779/83 | (9) | - |
| 693 | 17223/83 | 5 | Human | Skurnik lab strain collection | - |
| 709 | 18710/83 | 5 | Human | Skurnik lab strain collection | - |
| 944 | gk7500 | 5,27 | bt 1 | (10) | - |
| 1004 | JD E657 | 5,27 | aa^+^, ca^+^ | (11) | - |
| 1007 | JD E654 | 5,27 | aa^+^, ca^+^ | (11) | - |
| 96 | 590/80 | 6 | Human | (2) | + (0.5) |
| 92 | 189/80 | 6,30 | Human | (2) | - |
| 200 | 6737/80 | 6,30 | Human | (2) | - |
| 201 | 3604/80 | 6,30 | Human | (2) | - |
| 16 | 1309/80 | 6,31 | Human | (2) | - |
| 217 | 438/80 | 6,31 | Human | Skurnik lab strain collection | - |
| 28 | 22848/79 | 7,8 | Human | (2) | - |
| 698 | 17869/83 | 7,8 | Human | Skurnik lab strain collection | - |
| 252 | p310 | 8 | Lab strain | (2) | - |
| 277 | CDCA2635 | 8 | Human | (12) | - |
| 278 | TAMU | 8 | Human | (13) | - |
| 322 | WA+ | 8 | Human | (14) | - |
| 1 | 277/74 | 9 | Human | Skurnik lab strain collection | - |
| 21 | 4945/74 | 9 | Human | Skurnik lab strain collection | - |
| 54 | 767/73 | 9 | Human | Skurnik lab strain collection | - |
| 55 | 467/73 | 9 | Human | Skurnik lab strain collection | - |
| 1831 | 467/73-φR1-37-R | 9 | Spontaneous φR1-37 resistant derivative of 467/73 | (9) | - |
| 97 | 3672/74 | 9 | Human | Skurnik lab strain collection | - |
| 137 | 13752/73 | 9 | Human | Skurnik lab strain collection | - |
| 196 | Ruokola/71 | 9 | Human | (2) | + (0.006) |
| 1257 | Ruokola/71-c | 9 | pYV- derivative of Ruokola/71 | (2) | + (0.019) |
| 1901 | Ruokola/71-c-φR1-37-R | 9 | Spontaneous φR1-37 resistant derivative of Ruokola/71-c | Skurnik lab strain collection | - |
| 3066 | YeO9-OCR | 9 | *per*::kmR. Has not lost OC | (15) | + (1.217) |
| 4212 | YeO9-OC-1 | 9 | OC negative strain, KmGB inserted into OC gene cluster | (15) | + (0.013) |
| 4214 | YeO9-OC-P | 9 | Spontaneous φR1-37 resistant derivative of YeO9; partial OC loss | (15) | + (0.041) |
| 106 | 3102/80 | 10 | Human | (2) | - |
| 210 | 3788/80 | 10 | Human | (2) | - |
| 590 | 9613/83 | 41,(27)K1 | Human | Skurnik lab strain collection | - |
| 679 | 15712/83 | 14 | Human | (1) | - |
| 1830 | 80-EA-63-φR1-37-R | 21 | Spontaneous φR1-37 resistant derivative of 80-EA-63 | (9) | - |
| 704 | 18425/83 | 25,26,44 | Human | (1) | - |
| 1833 | 18425/83-φR1-37-R | 25,26,44 | Spontaneous φR1-37 resistant derivative of 18425/83 | (9) | - |
| 568 | 7104/83 | 35,36 | Human | (1) | - |
| 209 | 3229 | 50 | Human | (1) | - |
| 1829 | 3229-φR1-37-R | 50 | Spontaneous φR1-37 resistant derivative of 3229 | (9) | - |
| *Y. frederikseni* | | | | | |
| 502 | 38/83 | 48 | Human | (1) | - |
| 532 | 3400/83 | 16 | Human | (1) | - |
| *Y. intermedia* | | | | | |
| 1832 | 821/84-φR1-37-R | 52,54 | Spontaneous φR1-37 resistant derivative of 821/84 | (9) | - |
| *Y. pseudotuberculosis* | | | | | |
| 192 | 2812/79 | 1b | Human | (2) | - |
| 214 | 324/80 | 3 | Human | Skurnik lab strain collection | - |
| 245 | 1261/79 | 3 | Human | (2) | - |
| 407 | 677/82 | 1b | Human | Skurnik lab strain collection | - |

**References**

1. Skurnik M, Toivonen S. Identification of distinct lipopolysaccharide patterns among Yersinia enterocolitica and Y. enterocolitica-like bacteria. Biochemistry (Mosc). 2011;76(7):823-31.

2. Skurnik M. Lack of correlation between the presence of plasmids and fimbriae in Yersinia enterocolitica and Yersinia pseudotuberculosis. J Appl Bact. 1984;56:355-63.

3. Al-Hendy A, Toivanen P, Skurnik M. Lipopolysaccharide O side chain of Yersinia enterocolitica O:3 is an essential virulence factor in an orally infected murine model. Infect Immun. 1992;60:870-5.

4. Biedzka-Sarek M, Venho R, Skurnik M. Role of YadA, Ail, and lipopolysaccharide in serum resistance of Yersinia enterocolitica serotype O:3. Infect Immun. 2005;73(4):2232-44.

5. Noszczynska M, Kasperkiewicz K, Duda KA, Podhorodecka J, Rabsztyn K, Gwizdala K, et al. Serological characterization of the enterobacterial common antigen substitution of the lipopolysaccharide of Yersinia enterocolitica O : 3. Microbiology. 2015;161(Pt 1):219-27.

6. Happonen LJ, Pajunen MI, Jun JW, Skurnik M. BtuB-dependent infection of the T5-like Yersinia phage fR2-01. Viruses. 2021;13(11).

7. Pinta E, Li Z, Batzilla J, Pajunen M, Kasanen T, Rabsztyn K, et al. Identification of three oligo-/polysaccharide-specific ligases in Yersinia enterocolitica. Mol Microbiol. 2012;83(1):125-36.

8. Leon-Velarde CG, Happonen L, Pajunen M, Leskinen K, Kropinski AM, Mattinen L, et al. Yersinia enterocolitica-specific infection by bacteriophages TG1 and fR1-RT is dependent on temperature-regulated expression of the phage host receptor OmpF. Appl Environ Microb. 2016;82(17):5340-53.

9. Kiljunen S, Hakala K, Pinta E, Huttunen S, Pluta P, Gador A, et al. Yersiniophage fR1-37 is a tailed bacteriophage having a 270 kb DNA genome with thymidine replaced by deoxyuridine. Microbiology. 2005;151(Pt 12):4093-102.

10. Kapperud G, Skarpeid H-J, Solberg R, Bergan T. Outer membrane proteins and plasmids in different Yersinia enterocolitica serogroups isolated from man and animals. Acta path microbiol immunol scand Sect B. 1985;93:27-35.

11. Schiemann DA, Devenish JA. Relationship of HeLa cell infectivity to biochemical, serological, and virulence characteristics of Yersinia enterocolitica. Infect Immun. 1982;35:497-506.

12. Black RE, Jackson RJ, Tsai T, Medvesky M, Shayegani M, Feeley JC, et al. Epidemic Yersinia enterocolitica infection due to contaminated chocolate milk. New Engl J Med. 1976;298:76-9.

13. Gemski P, Lazere JR, Casey T. Plasmid associated with pathogenicity and calcium dependency of Yersinia enterocolitica. Infect Immun. 1980;27:682-5.

14. Carter PB. Pathogenicity of Yersinia enterocolitica for mice. Infect Immun. 1975;11:164-70.

15. Skurnik M, Biedzka-Sarek M, Lubeck PS, Blom T, Bengoechea JA, Perez-Gutierrez C, et al. Characterization and biological role of the O-polysaccharide gene cluster of Yersinia enterocolitica serotype O:9. J Bacteriol. 2007;189(20):7244-53.

**Supplementary Table S2.** Phage lytic activity in liquid cultures, tested by continuous OD_600_ measurement for 10h. The table presents normalized OD_600_ values of 42 *Yersinia* strains with and without phage infection, measured at final (10h) time point, in triplicates, the calculatyed averages and the ratio (with phage/without phage). The phage-sensitive strains are highlighted in blue.

| **Strain, serotype** | **With phage** | | | **Average** | **Without phage** | | | **Average** | **Ratio** |
| --- | --- | --- | --- | --- | --- | --- | --- | --- | --- |
| 6471/76-c, O:3 | 0.02 | 0.03 | 0.03 | 0.02 | 1.15 | 1.16 | 1.16 | 1.15 | 0.02 |
| 277/74, O:9 | 1.26 | 1.12 | 1.27 | 1.22 | 1.10 | 1.06 | 1.12 | 1.09 | 1.11 |
| 20373/79, O:3 | 0.91 | 0.91 | 0.89 | 0.90 | 1.05 | 1.15 | 1.04 | 1.08 | 0.84 |
| 5854, O:3 | 0.62 | 0.33 | 0.52 | 0.49 | 1.09 | 1.07 | 1.07 | 1.08 | 0.45 |
| 9568/79, O:3 | 0.17 | 0.15 | 0.16 | 0.16 | 1.17 | 1.10 | 0.43 | 0.90 | 0.18 |
| 1309/80, O:6.31 | 1.24 | 1.11 | 1.14 | 1.16 | 1.22 | 1.24 | 1.22 | 1.23 | 0.95 |
| 4945/74, O:9 | 1.15 | 1.20 | 1.24 | 1.20 | 1.13 | 1.22 | 1.19 | 1.18 | 1.02 |
| 22848/79, O:7.8 | 1.17 | 1.15 | 1.14 | 1.15 | 1.17 | 1.21 | 1.18 | 1.18 | 0.97 |
| 767/73, O:9 | 1.11 | 1.11 | 1.14 | 1.12 | 1.16 | 1.15 | 1.12 | 1.14 | 0.98 |
| 467/73, O:9 | 1.13 | 1.10 | 1.10 | 1.11 | 1.15 | 1.07 | 1.08 | 1.10 | 1.01 |
| 189/80, O:6.3 | 1.17 | 1.15 | 1.15 | 1.16 | 1.12 | 1.20 | 1.20 | 1.17 | 0.99 |
| 590/80, O:6 | 0.00 | 0.01 | 0.01 | 0.00 | 0.82 | 0.83 | 0.82 | 0.82 | 0.01 |
| 3672/74, O:9 | 0.61 | 0.63 | 0.63 | 0.62 | 0.64 | 0.64 | 0.63 | 0.63 | 0.99 |
| 3102/80, O:10 | 1.22 | 1.21 | 1.20 | 1.21 | 1.17 | 1.16 | 1.20 | 1.18 | 1.03 |
| 13752/73, O:9 | 1.19 | 1.22 | 1.22 | 1.21 | 1.17 | 1.13 | 1.10 | 1.13 | 1.07 |
| 2812/79, O:1b | 1.05 | 1.04 | 1.02 | 1.04 | 1.02 | 1.11 | 1.16 | 1.10 | 0.95 |
| 6737/80, O:6.3 | 1.15 | 1.14 | 1.14 | 1.14 | 1.10 | 1.09 | 1.09 | 1.09 | 1.04 |
| 3604/80, O:6.3 | 1.12 | 1.11 | 1.12 | 1.12 | 1.13 | 1.20 | 1.12 | 1.15 | 0.97 |
| 3229, O:50 | 1.17 | 1.17 | 1.17 | 1.17 | 1.03 | 1.01 | 1.00 | 1.01 | 1.15 |
| 3788/80, O:10 | 1.19 | 1.19 | 1.21 | 1.20 | 1.21 | 1.19 | 1.19 | 1.19 | 1.00 |
| 324/80, O:3 | 0.93 | 0.90 | 0.91 | 0.91 | 0.91 | 0.99 | 0.97 | 0.96 | 0.95 |
| 438/80, O:6.31 | 1.28 | 1.24 | 1.23 | 1.25 | 1.05 | 1.05 | 1.06 | 1.05 | 1.19 |
| 1261/79, O:3 | 1.13 | 1.13 | 1.15 | 1.14 | 1.10 | 1.09 | 1.04 | 1.07 | 1.06 |
| p310, O:8 | 0.90 | 0.90 | 0.89 | 0.90 | 0.81 | 0.81 | 0.79 | 0.80 | 1.12 |
| CDCA2635, O:8 | 1.18 | 1.19 | 1.22 | 1.19 | 1.07 | 1.00 | 0.97 | 1.02 | 1.18 |
| TAMU, O:8 | 1.17 | 1.22 | 1.19 | 1.20 | 1.14 | 1.18 | 1.18 | 1.17 | 1.02 |
| 404/81, O:16 | 1.03 | 0.98 | 0.96 | 0.99 | 1.06 | 1.08 | 1.12 | 1.08 | 0.91 |
| 1539 | 1.08 | 1.03 | 1.03 | 1.05 | 1.02 | 1.12 | 1.14 | 1.10 | 0.96 |
| wa+, O:8 | 0.97 | 0.97 | 0.92 | 0.96 | 0.91 | 0.91 | 0.92 | 0.92 | 1.04 |
| 317/82, O:16 | 1.04 | 0.96 | 0.96 | 0.99 | 0.97 | 0.94 | 0.99 | 0.97 | 1.02 |
| 677/82, O:1b | 0.78 | 0.76 | 0.75 | 0.76 | 0.61 | 0.74 | 0.73 | 0.69 | 1.10 |
| 38/83, O:48 | 1.17 | 1.20 | 1.19 | 1.19 | 1.12 | 1.12 | 1.14 | 1.13 | 1.05 |
| 3400/83, O:16 | 1.14 | 1.07 | 0.96 | 1.06 | 1.13 | 0.97 | 1.13 | 1.08 | 0.98 |
| 4367/83, K1 nt | 0.99 | 1.07 | 1.09 | 1.05 | 1.08 | 1.03 | 1.05 | 1.05 | 1.00 |
| 7104/83, O:35.36 | 1.08 | 1.07 | 0.93 | 1.03 | 1.00 | 1.04 | 1.05 | 1.03 | 1.00 |
| 9613/83, O:41(27)K1 | 1.11 | 0.91 | 1.04 | 1.02 | 1.03 | 1.05 | 1.02 | 1.03 | 0.99 |
| 14779/83, O:5 | 1.20 | 0.94 | 1.16 | 1.10 | 1.13 | 1.13 | 1.15 | 1.14 | 0.97 |
| 15712/83, O:14 | 1.18 | 1.15 | 1.15 | 1.16 | 1.09 | 1.07 | 1.03 | 1.06 | 1.09 |
| 17223/83, O:5 | 1.02 | 1.01 | 1.01 | 1.01 | 0.97 | 0.96 | 0.96 | 0.96 | 1.05 |
| 17869/83, O:7.8 | 1.09 | 0.93 | 0.93 | 0.98 | 1.02 | 1.02 | 1.04 | 1.03 | 0.96 |
| 18425/83, O:25,26,44 | 1.07 | 0.95 | 0.97 | 1.00 | 0.97 | 0.97 | 1.03 | 0.99 | 1.01 |
| 18710/83, O:5 | 1.05 | 0.96 | 1.03 | 1.01 | 0.89 | 0.90 | 0.94 | 0.91 | 1.11 |

|  | **Supplementary Table S3** Phage fMtkYen3-01 Functional Annotation | | | |  |  |
| --- | --- | --- | --- | --- | --- | --- |
|  |  |  |  |  |  |  |
| **Gene** | **Predicted function** | **Type** | **Start** | **End** | **Size bp** | **n AA of gene product** |
|  | source Bacteriophage sp. fMtkYen3 01 | source | 1 | 40415 | 40415 |  |
| g01 | hypothetical protein | CDS | 113 | 298 | 186 | 62 |
| g02 | hypothetical protein | CDS | 740 | 982 | 243 | 81 |
| g03 | hypothetical protein | CDS | 1019 | 1366 | 348 | 116 |
| g04 | hypothetical protein | CDS | 1359 | 1583 | 225 | 75 |
| g05 | ABC transporter | CDS | 1700 | 1870 | 171 | 57 |
| g06 | Serine/threonine-protein kinase | CDS | 1910 | 3130 | 1221 | 407 |
| g07 | DNA-directed RNA polymerase | CDS | 3188 | 5869 | 2682 | 894 |
| g08 | hypothetical protein | CDS | 5957 | 6490 | 534 | 178 |
| g09 | hypothetical protein | CDS | 6595 | 6729 | 135 | 45 |
| g10 | hypothetical protein | CDS | 6733 | 6987 | 255 | 85 |
| g11 | ATP-dependent DNA ligase | CDS | 7072 | 8187 | 1116 | 372 |
| g12 | hypothetical protein | CDS | 8189 | 8392 | 204 | 68 |
| g13 | hypothetical protein | CDS | 8560 | 8820 | 261 | 87 |
| g14 | Nucleotide kinase | CDS | 8820 | 9578 | 759 | 253 |
| g15 | hypothetical protein | CDS | 9571 | 9690 | 120 | 40 |
| g16 | Host RNA polymerase inhibitor | CDS | 9694 | 9885 | 192 | 64 |
| g17 | hypothetical protein | CDS | 9887 | 10267 | 381 | 127 |
| g18 | Single-stranded DNA-binding protein | CDS | 10334 | 11041 | 708 | 236 |
| g19 | Endonuclease I | CDS | 11108 | 11563 | 456 | 152 |
| g20 | Endolysin | CDS | 11568 | 12023 | 456 | 152 |
| g21 | DNA primase/helicase | CDS | 12104 | 13807 | 1704 | 568 |
| g22 | hypothetical protein | CDS | 13910 | 14143 | 234 | 78 |
| g23 | hypothetical protein | CDS | 14154 | 14456 | 303 | 101 |
| g24 | hypothetical protein | CDS | 14529 | 14933 | 405 | 135 |
| g25 | DNA-directed DNA polymerase | CDS | 14951 | 17065 | 2115 | 705 |
| g26 | DUF2675 domain-containing protein | CDS | 17090 | 17398 | 309 | 103 |
| g27 | Fusion protein | CDS | 17398 | 17607 | 210 | 70 |
| g28 | hypothetical protein | CDS | 17604 | 17891 | 288 | 96 |
| g29 | Exonuclease | CDS | 17888 | 18805 | 918 | 306 |
| g30 | hypothetical protein | CDS | 19016 | 19273 | 258 | 86 |
| g31 | DUF5476 domain-containing protein | CDS | 19284 | 19562 | 279 | 93 |
| g32 | Phage virion assembly protein | CDS | 19578 | 19844 | 267 | 89 |
| g33 | Head-to-tail connector protein | CDS | 19858 | 21465 | 1608 | 536 |
| g34 | Capsid and scaffolding protein | CDS | 21529 | 22446 | 918 | 306 |
| g35 | Major capsid protein | CDS | 22595 | 23638 | 1044 | 348 |
| g36 | Non-contractile tail tubular protein | CDS | 23843 | 24433 | 591 | 197 |
| g37 | Non-contractile tail tubular protein | CDS | 24447 | 26819 | 2373 | 791 |
| g38 | Phage scaffold protein | CDS | 26888 | 27298 | 411 | 137 |
| g39 | Internal virion protein | CDS | 27301 | 27876 | 576 | 192 |
| g40 | Internal virion protein | CDS | 27901 | 30150 | 2250 | 750 |
| g41 | Peptidoglycan lytic exotransglycosylase | CDS | 30165 | 34124 | 3960 | 1320 |
| g42 | Non-contractile tail fiber protein | CDS | 34180 | 35763 | 1584 | 528 |
| g43 | Phage holin, class II | CDS | 35785 | 35991 | 207 | 69 |
| g44 | Phage terminase small subunit | CDS | 35984 | 36250 | 267 | 89 |
| g45 | Rz-like lysis protein | CDS | 36351 | 36806 | 456 | 152 |
| g46 | hypothetical protein | CDS | 36806 | 37285 | 480 | 160 |
| g47 | Phage terminase large subunit | CDS | 37396 | 39147 | 1752 | 584 |
| g48 | HNH homing endonuclease | CDS | 39158 | 39607 | 450 | 150 |
| g49 | Methyltransferase type 11 | CDS | 39854 | 40009 | 156 | 52 |
|  |  |  |  |  |  |  |

**Supplementary Table S4** Nucleotide sequence similarities between fMtkYen3-01 and most closely related phages, generated by BLASTn

| **Phage** | **Coverage (%)** | **Identity (%)** | **Whole genome identity %** | **E value** | **Accession no.** |
| --- | --- | --- | --- | --- | --- |
| Yersinia phage fPS-59 | 82% | 90.65% | 74 | 0 | [NC047935.1](https://www.ncbi.nlm.nih.gov/nucleotide/NC_047935.1?report=genbank&log$=nucltop&blast_rank=1&RID=5XEP8D25013) |
| [Yersinia phage fPS-50](https://www.ncbi.nlm.nih.gov/Taxonomy/Browser/wwwtax.cgi?id=2052750) | 79% | 89.93% | 71 | 0 | [LT961843.1](https://www.ncbi.nlm.nih.gov/nucleotide/LT961843.1?report=genbank&log$=nucltop&blast_rank=8&RID=5XEP8D25013) |
| [Yersinia phage fPS-10](https://www.ncbi.nlm.nih.gov/Taxonomy/Browser/wwwtax.cgi?id=2052933) | 79% | 89.93% | 71 | 0 | [LT962907.1](https://www.ncbi.nlm.nih.gov/nucleotide/LT962907.1?report=genbank&log$=nucltop&blast_rank=6&RID=5XEP8D25013) |
| [Yersinia phage fPS-86](https://www.ncbi.nlm.nih.gov/Taxonomy/Browser/wwwtax.cgi?id=2052757) | 79% | 89.93% | 71 | 0 | [LT961842.1](https://www.ncbi.nlm.nih.gov/nucleotide/LT961842.1?report=genbank&log$=nucltop&blast_rank=9&RID=5XEP8D25013) |
| [Yersinia phage fPS-19](https://www.ncbi.nlm.nih.gov/Taxonomy/Browser/wwwtax.cgi?id=2052747) | 79% | 89.91% | 71 | 0 | [LT961838.1](https://www.ncbi.nlm.nih.gov/nucleotide/LT961838.1?report=genbank&log$=nucltop&blast_rank=10&RID=5XEP8D25013) |
| [Yersinia phage fPS-21](https://www.ncbi.nlm.nih.gov/Taxonomy/Browser/wwwtax.cgi?id=2052748) | 79% | 89.93% | 71 | 0 | [LT961844.1](https://www.ncbi.nlm.nih.gov/nucleotide/LT961844.1?report=genbank&log$=nucltop&blast_rank=7&RID=5XEP8D25013) |
| [Yersinia phage fPS-54-ocr](https://www.ncbi.nlm.nih.gov/Taxonomy/Browser/wwwtax.cgi?id=2052753) | 80% | 90.20% | 72 | 0 | [NC047937.1](https://www.ncbi.nlm.nih.gov/nucleotide/NC_047937.1?report=genbank&log$=nucltop&blast_rank=2&RID=5XEP8D25013) |
| [Yersinia phage fPS-53](https://www.ncbi.nlm.nih.gov/Taxonomy/Browser/wwwtax.cgi?id=2052752) | 77% | 90.20% | 69 | 0 | [NC047936.1](https://www.ncbi.nlm.nih.gov/nucleotide/NC_047936.1?report=genbank&log$=nucltop&blast_rank=4&RID=5XEP8D25013) |
| [Yersinia phage fPS-89](https://www.ncbi.nlm.nih.gov/Taxonomy/Browser/wwwtax.cgi?id=2052744) | 79% | 90.20% | 71 | 0 | [LT961841.1](https://www.ncbi.nlm.nih.gov/nucleotide/LT961841.1?report=genbank&log$=nucltop&blast_rank=5&RID=5XEP8D25013) |
| [Yersinia phage fPS-85](https://www.ncbi.nlm.nih.gov/Taxonomy/Browser/wwwtax.cgi?id=2052756) | 79% | 90.20% | 71 | 0 | [LT962380.1](https://www.ncbi.nlm.nih.gov/nucleotide/LT962380.1?report=genbank&log$=nucltop&blast_rank=3&RID=5XEP8D25013) |
